# A response to “Should children under 5 and those with constipation be overlooked from ARFID research?”

**DOI:** 10.1016/j.eclinm.2024.102668

**Published:** 2024-06-04

**Authors:** Javier Sanchez-Cerezo, Josephine Neale, Nikita Julius, Tim Croudace, Richard M. Lynn, Lee D. Hudson, Dasha Nicholls

**Affiliations:** a Division of Psychiatry, Department of Brain Sciences, Imperial College London, London, UK; b Department of Psychiatry, Hospital Universitario Puerta de Hierro, Majadahonda, Madrid, Spain; c Priory Hospital Ticehurst House, Ticehurst, East Sussex, UK; d School of Health Sciences, University of Dundee, Dundee, UK; e Institute of Child Health, University College London, London, UK

Thank you for your time and effort of writing a letter raising some concerns about our paper entitled ‘Subtypes of avoidant/restrictive food intake disorder in children and adolescents: a latent class analysis’. We provide detailed responses to the raised concerns below.

In our study, we identified children and adolescents with ARFID attending secondary care across the UK and Ireland using active surveillance methodology in collaboration with the British Paediatric Surveillance Unit (BPSU) and the Child and Adolescent Psychiatry Surveillance System (CAPSS). As explained in the paper, these paediatric surveillance units work by surveying consultant paediatricians and child and adolescent psychiatrists who see patients from a different age range (i.e., child and adolescent psychiatrists see patients from 5 years of age). Picky or fussy eating is common in younger children,^1^ and therefore may be more difficult to differentiate from cases of ARFID and carries the risk of overreporting in children under 5 years of age. In addition, our study had as a secondary objective to compare rates of ARFID with other countries such as Canada, where another surveillance study of ARFID in children and adolescents aged 5–18 years was conducted.^2^ We agree that ARFID can be diagnosed across the lifespan^3^ and in the future, further surveillance studies of ARFID including younger children should be done which will help to better understand early presentations of this disorder.

According to the DSM-5,^3^ an eating disturbance categorized as ARFID should not be attributable to a concurrent medical condition or better explained by another mental disorder. Even so, if the disturbance does occur in the context of another condition or disorder, it may be categorized as ARFID if the severity exceeds that which is commonly seen and warrants additional clinical attention. Constipation and other conditions such as food allergies were not absolute reasons for exclusion if the severity of eating disturbance exceeded that routinely associated with the condition and warranted additional clinical attention. We specified this in the footnote for Table 1 in the paper.^4^ We agree that patients with ARFID often have comorbidities with gastrointestinal symptoms.^5^ Indeed, unpublished analysis in our sample shows that almost 22% of it had constipation.

## Contributors

Javier Sanchez-Cerezo: conception and design of the work, drafting the work.

Josephine Neale: drafting and critically reviewing the work.

Nikita Julius: critically reviewing the work.

Tim Croudace: critically reviewing the work.

Richard M Lynn: drafting and critically reviewing the work.

Lee D Hudson: drafting and critically reviewing the work.

Dasha Nicholls: drafting and critically reviewing the work. Supervision.

All authors approved the final version of the letter. All authors accept responsibility to submit for publication.

## Declaration of interests

No conflicts of interest to declare.
